# Epigenetic Alterations in Pancreatic Cancer Metastasis

**DOI:** 10.3390/biom11081082

**Published:** 2021-07-22

**Authors:** Sarah S. Wang, Jihao Xu, Keely Y. Ji, Chang-Il Hwang

**Affiliations:** 1 Department of Microbiology and Molecular Genetics, College of Biological Sciences, University of California Davis, Davis, CA 95616, USA; saswang@ucdavis.edu (S.S.W.); jihxu@ucdavis.edu (J.X.); kyji@ucdavis.edu (K.Y.J.); 2 Biochemistry, Molecular, Cellular and Developmental Biology Graduate Group, University of California Davis, Davis, CA 95616, USA; 3 Comprehensive Cancer Center, University of California Davis, Sacramento, CA 95817, USA

**Keywords:** pancreatic cancer, epigenetics, metastasis, DNA methylation

## Abstract

Pancreatic cancer is the third leading cause of cancer-related deaths in the United States. Pancreatic ductal adenocarcinoma (PDA) is the most common (90%) and aggressive type of pancreatic cancer. Genomic analyses of PDA specimens have identified the recurrent genetic mutations that drive PDA initiation and progression. However, the underlying mechanisms that further drive PDA metastasis remain elusive. Despite many attempts, no recurrent genetic mutation driving PDA metastasis has been found, suggesting that PDA metastasis is driven by epigenetic fluctuations rather than genetic factors. Therefore, establishing epigenetic mechanisms of PDA metastasis would facilitate the development of successful therapeutic interventions. In this review, we provide a comprehensive overview on the role of epigenetic mechanisms in PDA as a critical contributor on PDA progression and metastasis. In particular, we explore the recent advancements elucidating the role of nucleosome remodeling, histone modification, and DNA methylation in the process of cancer metastasis.

## 1. Introduction

Pancreatic cancer is the third leading cause of cancer-related deaths in the United States. These poor survival outcomes are primarily because pancreatic cancer is often asymptomatic in its early stages, making early diagnoses difficult. The five-year survival rate for pancreatic cancer is 10%, the lowest among common cancers, and pancreatic cancer is expected to surpass colorectal cancer as the second leading cause of cancer-related deaths by 2030 [1,2]. Of pancreatic cancer types, pancreatic ductal adenocarcinoma (PDA) is the most common and aggressive, comprising 90% of pancreatic cancer patients [3]. Despite the fact that recent advances in first-line chemotherapy, such as FOLFIRINOX or gemcitabine/nab-paclitaxel, survival benefits for PDA patients remain modest [4,5]. As a consequence, significant effort has been made to understand the progression of the disease.

**Figure 1:**
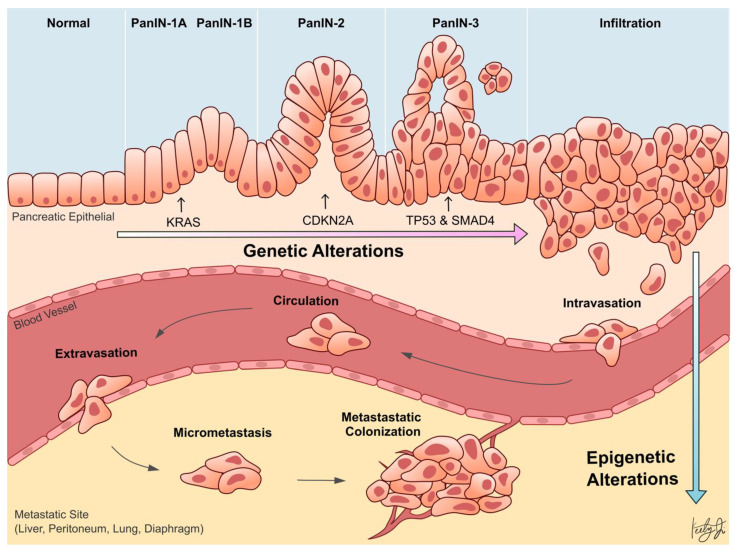
Schematic illustration showing pancreatic ductal adenocarcinoma (PDA) progression from the normal pancreas, pancreatic intraepithelial neoplasia (PanIN), and metastasis. Early pancreatic carcinogenesis is driven by genetic alterations in *KRAS*, *CDKN2A*, *TP53*, and *SMAD4* (**top**). During metastasis, PDA cells penetrate the blood vessel (intravasation), circulate through the bloodstream, invade into the metastatic site (extravasation), and colonize to form a secondary malignant tumor. The process of pancreatic cancer metastasis is facilitated by epigenetic alterations (**bottom**).

Whole-genome sequencing technologies have undoubtedly revealed that PDA is a disease that arises from genetic aberrations. Notably, the initiating genetic event in over 90% of PDA cases is a gain-of-function mutation of *KRAS* in acinar or ductal cells, which results in the formation of pancreatic lesions called pancreatic intraepithelial neoplasia (PanIN). Subsequent loss-of-function mutations or deletions in tumor suppressor genes, such as *TP53, SMAD4*, and *CDKN2A*, cooperate with *KRAS* mutation to drive tumor formation and further exacerbate the disease progression (Figure 1) [6,7]. In addition to genetic aberrations, it has become increasingly clear over the last two decades that epigenetic alterations also promote the progression of almost every type of cancer [8,9,10,11]. Epigenetic mechanisms regulate gene transcription, and the proper functioning of these mechanisms is essential for normal development and tissue differentiation. When these mechanisms are aberrantly altered in cancer cells, they can silence tumor suppressor genes or promote the expression of oncogenes to confer advantageous adaptations of the cancer cells, such as increased survival and proliferation, leading to aggressive cell phenotypes and metastasis.

This review seeks to comprehensively assess the current progress regarding the role of epigenetic alterations in PDA progression and metastasis. Specifically, recent studies investigating the role of alterations in epigenetic regulators, histone modifications, chromatin accessibilities, and DNA methylation in PDA are highlighted (Table 1).

## 2. Genetic Alterations in Epigenetic Regulators

While *KRAS, TP53, SMAD4,* and *CDKN2A* driver mutations are core to early PDA progression, there is a vast genetic heterogeneity among PDA patients, harboring a range of less frequent genetic mutations that facilitate carcinogenesis [6,7]. For one, around 10% of PDA cases belong to familial pancreatic cancer and are commonly affected by germline pre-mature truncating variant (PTV) mutations in genes related to the DNA repair pathways (e.g., *BRCA1/2, ATM,* and *PALB2*), which have been predicted to inactivate the proteins [6,12]. Interestingly, a subset of these patients also have germline PTV mutations in epigenetic regulators (e.g., *TET2*, *DNMT3A,* and *ASXL1*) [12], suggesting that aberrant changes to the epigenome are important in predisposing individuals to PDA by altering the transcriptional profile of cells.

In addition to the germline mutations in PDA, whole exome and genome sequencing revealed that a significant percentage of patients with PDA have somatic mutations in epigenetic regulators and chromatin remodeling complexes (e.g., *ARID1A/B*, *PBRM1*, *MLL2/3/4*, *KDM6A*, *SMARCA2/4*) [6,7]. Furthermore, somatic mutations in SWI/SNF complex regulators (e.g., *ARID1A*) and inactivation of histone modification enzymes (e.g., *MLL3*, *MLL5*, *KDM6A*) frequently occurred in conjunction with oncogenic *KRAS* in sleeping beauty transposon insertional mutagenesis screens [13,14]. Indeed, Mann et al. found that 100% of the tumors in this screen harbored one or more mutations in genes coding for histone-modifying enzyme [13]. These mutations cooperated with oncogenic KRAS to promote PDA progression, suggesting that alterations to the epigenome are important for driving PDA progression [13]. Together these findings highlight the significance of epigenetic regulation in pancreatic cancer progression.

Despite our firm understanding of the genetic alterations that underlie PDA progression, there is limited understanding of the genetic drivers of PDA metastasis. To this day, there is no known recurrent mutation that drives this metastatic process [21,22,23,24]. This suggests that epigenetic mechanisms, rather than genetic, are driving PDA metastasis (Figure 1). Perturbed epigenetic programs, including transcription factor (TF)-mediated histone modifications, chromatin remodeling, DNA methylation patterns, and subsequently altered transcriptional programs, are emerging mechanisms of PDA progression and metastasis [25].

## 3. Chromatin Accessibility and Metastasis

**Figure 2:**
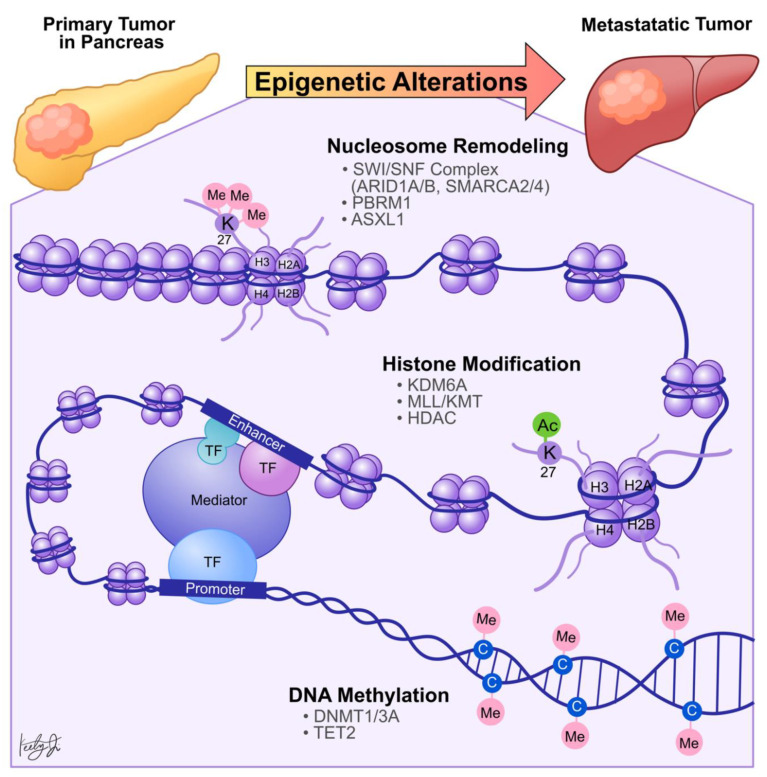
Summary of the epigenetic mechanisms behind PDA metastasis. Metastatic PDA tumors have aberrant epigenetic profiles that are different from PDA primary tumors. Nucleosome remodeling and histone modification (e.g., acetylation of histone 3 lysine 9) increase chromatin accessibility, allowing for transcription factor binding and gene transcription. On the other hand, DNA methylation of CpG islands leads to gene repression. These processes are mediated by epigenetic regulators, some of which are noted in the figure.

Chromatin is composed of nucleosomes, which are formed by DNA wrapped around canonical histone molecules, including H2A, H2B, H3, and H4 (Figure 2) [26]. There are two distinct chromatin states: open or euchromatin, and closed or heterochromatin. In the heterochromatin state, nucleosomes are highly condensed, preventing TF binding and subsequent RNA polymerase recruitment [27]. Therefore, to initiate gene transcription, both *cis* (gene promoters and enhancers) and *trans* (TFs and RNA polymerases) regulatory elements interact in a spatial and temporal manner to establish the euchromatin architecture, allowing the recruitment of transcriptional machinery that favors transcription initiation. To achieve this, *trans* pioneer TFs first bind to nucleosomal DNA within the heterochromatin and recruit chromatin remodeling enzymes to remodel nucleosomes and expose the *cis* elements, such as enhancers [28]. Enhancers are distal elements independent of the distance and orientation of the targeted genes [29]. In addition, enhancers contain unique DNA sequence motifs to recruit specific TFs and co-activators or co-repressors, and the gene transcription activities are determined by the summation of all the co-regulators [30].

In the context of cancer metastasis, Denny et al. compared chromatin accessibility between primary and metastatic small cell lung cancer (SCLC) using Assay for Transposase-Accessible Chromatin followed by sequencing (ATAC-seq) [31]. The study showed that aggressive metastatic SCLC overexpresses embryonic developmental TF NF1 to remodel nucleosomes around the TF-binding enhancers and establish the euchromatin architecture. In turn, the open chromatin architecture allows the upregulation of transcription programs related to axon guidance, neuron development, cell-cell adhesion, migration, and differentiation. Together, evidenced by in vitro cell migration and colony formation assays and in vivo subcutaneous and intravenous transplantation assays, these programs promote proliferation and migratory abilities of the cancer cell in vitro and metastasis in vivo [31]. In pancreatic cancer, Dhara et al. used ATAC-seq to analyze chromatin accessibility of surgically resected PDA between patients with disease-free survival (DSF) less than one year (cancer recurrent/metastases group) and patients with DSF greater than one year (cancer non-recurrent/non-metastases group) and found 1092 differentially accessible chromatin peaks between the PDA recurrent and non-recurrent patients [15]. Subsequent computational TF motif analysis identified 61 TFs with binding motifs within these chromatin regions. These TFs included tumor-promoting ZKSCAN1 from the open chromatin regions of metastases patients and tumor suppressor HNF1B from the open chromatin regions of non-metastases patients [15]. Together, these studies demonstrated that cancer cells, including PDA, can remodel chromatin landscape and accessibility to recruit or prevent TF binding as a mechanism to initiate tumor metastasis. Furthermore, detection of the disrupted chromatin landscapes in tumor biopsy samples could potentially be used for PDA prognostic predictions in clinical settings.

## 4. Transcription Factor-Mediated Histone Modification and Metastasis

Several histone post-translational modifications (PTMs) co-occur during chromatin remodeling and gene transcription, which can be used as indicators of transcriptional activities. In general, active promoters are marked by dual H3K27 acetylation (H3K27ac) and H3K4 trimethylation (H3K4me3), while active enhancers are marked by dual H3K27ac and H3K4 monomethylation (H3K4me1) [32]. Conversely, histone H3K9 and H3K27 methylation are used to indicate repressive gene transcription [33]. These histone PTMs alter biochemical properties of the chromatin, not only leading to the formation of euchromatin or heterochromatin, but also the sequestering or docking of effector enzymes, such as histone acetylase, deacetylase, methyltransferase, and demethylase [34].

Dysregulation in histone PTMs, in conjunction with the recruitment of effector chromatin remodelers, modifies chromatin architecture, leading to aberrant gene activation or repression, which contributes to cancer metastasis (Figure 2). For example, by comparing primary PDA tumors to matched distant lung and proximal peritoneum metastases, McDonald et al. used chromatin immunoprecipitation followed by sequencing (ChIP-seq) to demonstrate that global alterations of H3K9me2/3 and H3K27ac may contribute to aggressive tumor phenotypes [35]. Specifically, ChIP-seq showed that H3K9 methylation levels are reduced at Large Organized Chromatin K9-modified (LOCK) heterochromatin regions in distant metastases compared to their matched primary tumors [35], suggesting that transcription activities of certain genes within these regions (LOCK genes) are upregulated during PDA metastasis. Indeed, using RNA-seq and ChIP-seq analysis, the study showed that decreased H3K9me2 and H3K27me3 and increased H3K27ac occupancies at gene promoters in LOCK regions is positively correlated with expression of the associated genes in distant metastases [35]. Furthermore, subsequent gene ontology analysis revealed that the reprogrammed LOCK regions contain genes related to cellular differentiation and morphogenesis, epithelial-to-mesenchymal transitions, cell adhesion, and migration [35]. This suggests that a histone modification-mediated epigenetic switch from heterochromatin to euchromatin state is associated with cellular transformation, which promotes aggressive tumor phenotypes and facilitates PDA tumor-to-metastasis transitions.

Histone modification-dependent epigenetic landscape reprogramming can be carried out by TFs through first targeting nucleosomal DNA and then recruiting histone and chromatin remodeling enzymes [28]. In the context of pancreatic cancer, we identified that the developmental Forkhead family TF FOXA1 drives enhancer landscape reprogramming during PDA tumor-to-metastasis transition [16]. To dissect the molecular mechanisms of enhancer activation/inactivation during PDA metastasis, we developed 3D organoid culture using PDA cells collected from the primary tumors and matched metastatic lesions derived from the *Kras*^+/LSL-G12D^; *Trp53*^+/LSL-R172H^; *Pdx1-Cre* (KPC) PDA mouse model [36]. The organoid culture model of PDA preserves the unique biological characteristics of normal, PanIN, tumor, and metastases lesions. In addition, this model can be used for direct biochemical comparisons during each stage of the disease progression [36]. H3K27ac ChIP-seq analysis revealed 857 regions with increased H3K27ac occupancy (*GAIN* region) in the metastases organoids compared to the normal, PanIN, and tumor organoids [16], suggesting that the dysregulation of H3K27ac landscape within these enhancer regions could be responsible for PDA progression and metastasis. Combining RNA-seq and TF motif analysis, we then identified that FOXA1 activates *GAIN* region enhancers by increasing H3K27ac occupancy in the primary PDA. *In vitro*, overexpression of FOXA1 in primary PDA tumor cells activated foregut developmental genes that promoted anchorage-independent cell growth and invasion in sphere-formation and Matrigel invasion assays, respectively. *In vivo*, overexpression of FOXA1 contributed to overall PDA progression and metastasis in tail vein injection and organoid transplantation experiments [16]. This work demonstrated that PDA cells could repurpose FOXA1 to activate enhancers of developmental gene programs [37], promote anchorage-independent growth, and induce branching morphogenesis of the epithelial cells [38,39]. Furthermore, upregulation of FOXA1 in PDA cells promotes aggressive cell phenotypes, such as proliferation, invasion, and migration, allowing cells to better withstand stressful conditions during metastasis. In support of our work, Kim et al. discovered that *FOXA1* gene transcription is enhanced by missense mutations of p53 (p53^R172H^, p53^R245W^, and p53^R270H^) that directly bind to the *FOXA1* promoter and induce oncogenic KRAS activation of cyclic AMP responsive element binding protein 1 (CREB1) [40]. In turn, FOXA1 promotes β-catenin stabilization and subsequently activates canonical WNT transcriptional programs to promote anchorage-independent cell growth, proliferation, and metastasis [40,41]. Together, these studies demonstrated that PDA cells could reprogram the epigenetic landscape and subsequent transcription programs through (1) recruiting TFs, (2) altering chromatin architectures through histone modifications, and (3) recruiting transcription co-activators (i.e., mutant p53 and CREB1) to sustain their growth, differentiation, and metastasis (Figure 2).

## 5. Transcription Factor-Mediated Enhancer Regulation in Aggressive PDA Molecular Subtype Differentiation

PDA can be categorized into four subtypes based on the gene transcriptional programs identified by Bailey et al. They are the squamous, pancreatic progenitor, immunogenic, and aberrantly differentiated endocrine exocrine (ADEX) subtype [42]. The two most common are the squamous and the progenitor subtype [42,43,44]. The progenitor subtype of PDA expresses pancreatic endoderm lineage-specific TFs, including PDX1, GATA6, FOXA2/3, HNF1A/B, and HNF4A/G [42,43]. In contrast, the squamous subtype of PDA represses the endoderm lineage-specific TFs through DNA hypermethylation at the gene promoters [42]. In addition, the squamous subtype upregulates TF p63 expression and is often associated with poor PDA patient prognosis [42,44]. Somerville et al. demonstrated that aberrant expression of p63 reprograms the enhancer landscape of PDA, leading to the upregulation of squamous transcriptional programs to promote tumor growth and metastatic potential, which is evident by primary tumor size and number of metastatic lesions in the xenograft transplantation model [17]. Similar to FOXA1-dependent enhancer reprogramming, this study found that p63 increases H3K27ac occupancy at the enhancers of squamous lineage genes, resulting in increased transcriptions of genes including *KRT5/6*, *TRIM29*, and *PTHLH*. Together, the squamous transcriptional program governed by epigenetic mechanisms promotes aggressive PDA phenotypes in vivo [17].

Mutations in epigenetic modulators, including histone H3K27me2/3-specific lysine demethylase 6A (KDM6A) [42,45], are commonly found in PDA squamous subtypes. Therefore, the cancer cell could potentially utilize or silence these epigenetic modulators to acquire metastatic traits. For example, given that at least 18% of PDA patient carries KDM6A mutations [7], which are associated with the squamous molecular subtype, Andricovich et al. found that loss of KDM6A in PDA can directly induce the squamous identity by upregulating the expressions of specific TF encoding genes, including *p63*, *ZEB1*, *RUNX3*, and *MYC* [45]. Mechanistically, loss of KDM6A allowed histone type 2 lysine methyltransferases (KMT2) to occupy and activate enhancers of squamous differentiation-promoting genes (squamous elements), which is evident by increased H3K4me1 and KMT2D occupancies at the squamous elements [45]. KMT2 enzyme families are histone H3K4-specific methyltransferases that mark active gene enhancers with H3K4me1 [32,46]. Interestingly, KDM6A has been shown to partner with KMT2 to form the COMPASS (complex of proteins associated with Set1)-like complex [47], suggesting that PDA utilizes the loss of KDM6A to relieve enhancer repressions through COMPASS-like complex-dependent histone H3K4 modifications. In turn, the activated enhancers facilitate the expression of the squamous lineage genes to gain metastatic potential. Together, these studies showed that PDA cells could remodel the epigenetic landscape by repressing key epigenetic modulators to upregulate TFs that drive squamous PDA transcriptional programs. These programs favor cellular adaptations that promote an aggressive PDA phenotype and metastasis.

## 6. DNA Methylation and Metastasis

Another epigenetic mechanism that is likely contributing to PDA metastasis is DNA methylation (Figure 2). DNA methylation is the process by which a methyl group is added to the 5′ carbon of cytosines, primarily at CpG sites where the cytosine is followed by guanine [48]. This methylation is catalyzed by DNA methyltransferases (DNMTs) and removed by ten-eleven translocase (TET) enzymes or inhibition of the maintenance methylase, DNMT1 [49,50]. At promoters and enhancers, DNA methylation is negatively correlated with gene expression as the methylation can inhibit the binding of transcription factors binding, promote the binding of transcriptional repressor complexes, and encourage a closed, heterochromatin state [51,52,53]. In intergenic regions, DNA methylation is positively correlated with gene expression, but the role and regulations of this methylation are still poorly understood [54,55]. Numerous studies have shown that DNA methylation is dysregulated in virtually every cancer, with cancer cells exhibiting extensive differential methylation compared to normal cells [56,57,58].

In PDA, aberrant DNA methylation has been widely documented. Early on, these studies involved methylated CpG island amplification (MCA) followed by microarray sequencing. More recently, bisulfite treatment paired with large microarray platforms or next-generation sequencing, such as reduced-representative bisulfite sequencing (RRBS) or whole-genome bisulfite sequencing (WGBS), have been used to assess the DNA methylome at base-pair resolution. Using these methods, DNA methylation in PDA has been correlated with several disease outcomes and histopathological phenotypes. For example, Thompson et al. identified 17,251 CpG sites that are negatively associated with survival outcome and 3256 sites that are positively associated with survival outcome in a comparison of RRBS data from a small cohort of PDA patient tissues and adjacent normal pancreas tissues [59]. Similarly, Mishra et al. identified 406 promoter methylation loci associated with survival in an analysis of 450K array methylation data from the 154 PDA samples in The Cancer Genome Atlas pancreatic cancer patient database (TCGA-PAAD) [60]. Unsupervised clustering of TCGA-PAAD samples based on the differentially methylated CpG sites resulted in three distinct clusters of patient samples [61]. These clusters were each enriched with a different tumor grade, indicating that DNA methylation can be used to estimate the histopathological stage of PDA tumors [61]. In an analysis of both TCGA-PAAD transcriptome and DNA methylome data, unsupervised subtyping of TCGA-PAAD samples based on genes whose expression was significantly correlated with methylation expression patterns was performed [62]. Interestingly, this analysis identified five subtypes, four of which correspond to the molecular subtypes identified by Bailey et al. (i.e., squamous, pancreatic progenitor, immunogenic, and aberrantly differentiated endocrine exocrine [ADEX]), and the last unique subtype was enriched for tumor microenvironment related genes [42,62]. Together, these data suggest that aberrant DNA methylation is associated with aggressive PDA phenotypes.

To identify pathways that may be involved in DNA methylation-mediated PDA aggressiveness, gene ontology and/or pathway analysis is often performed on differentially methylated genes in PDA. In the Thompson et al. study, CpG sites with a negative correlation between methylation and survival rate were associated with pancreas-specific development genes [59]. Normally, pancreas-specific development genes are only active in early embryonic stages, but reactivation of these genes during PDA is common [63,64]. Differential methylation of pancreas development genes has also been noted in the TCGA-PAAD dataset, suggesting that reactivation of the embryonic pancreas development program in PDA is epigenetically regulated [61]. Other differentially methylated genes found in the TCGA-PAAD dataset were enriched for cancer-related pathways, including MAPK, Rap1, and calcium signaling [60,61]. In addition, core signaling pathways that are commonly altered in PDA, such as Wnt/Notch signaling, apoptosis, cell-cycle regulation, and cell adhesion, were enriched in aberrantly methylated genes found in the analysis of TCGA-PAAD database as well as a separate bisulfite microarray study of 167 PDA and 29 adjacent normal pancreata conducted by Nones et al. [20,61,65]. Interestingly, Nones et al. also observed that stellate cell activation genes were often hypomethylated and therefore, likely downregulated in PDA [20]. In support of this, Espinet et al. discovered that stellate cells exposed to conditioned media derived from high-interferon (IFN) signature patient tissues showed an increased stellate cell growth in vitro and tumor formation in vivo [66]. Because the IFN pathway is involved in anti-viral defense and activated stellate cells are involved in ECM remodeling, this pathway may be activating and reprogramming stellate cells to produce a pro-inflammatory stroma that facilitates tumor growth. Hypermethylation of homeobox genes, which encode key transcription factors of embryonic development, was also commonly detected in several PDA methylome studies [53,61,66,67]. This provides additional evidence that PDA tumors reactivate developmental pathways via epigenetic mechanisms to promote metastatic characteristics. Overall, these studies suggest that processes commonly implicated in cancer aggressiveness and metastasis, such as apoptosis, cell-cycle regulation, and development pathways, are heavily influenced by aberrant DNA methylation in PDA.

Aberrant methylation of several individual genes and their association with worse survival outcomes have also been documented. Most of these genes have been reported to have hypermethylated promoters. For example, Sato et al. showed that the low expression of *TFPI-2*, which encodes a negative regulator of pro-metastasis extracellular matrix degradation, is frequently seen in both PDA cell lines and primary tumors and is associated with hypermethylation of the *TFPI-2* promoter [18]. Restoration of TFPI-2 in the PDA cell lines reduced proliferation and migratory potential [18]. Likewise, *RELN*, which encodes a critical regulator of neuronal migration, is commonly hypermethylated and silenced in pancreatic cancer [19]. Furthermore, low expression of *RELN* was significantly associated with worse survival outcomes, and siRNA knockdown of RELN in RELN-expressing pancreatic cancer cells enhanced cell motility, invasion, and colony formation [19]. Nevertheless, promoter hypomethylation of certain genes has also been implicated in a worse prognosis. For example, hypomethylation of *MET*, whose aberrant expression promotes metastasis, and *ITGA2*, which is involved in cell adhesion, correlated with increased mRNA expression and associated with poor survival in PDA by Nones et al. [20]. Thus, aberrant promoter methylation likely contributes to the aggressive nature of PDA by altering the expression of genes such as *TFPI-2*, *RELN*, *MET*, and *ITGA2*.

While the above studies provide a strong indication that aberrant DNA methylation in PDA likely contributes to metastasis, it is important to note that many of these studies are largely association-based and therefore do not functionally implicate genes or pathways in the process. Studies with functional experiments have shown that altering the expression of aberrantly methylated genes with loss-of-function or gain-of-function approaches affects metastatic potential *in vitro*, but analogous experiments in vivo are lacking. Furthermore, no study has directly shown the consequences of altered DNA methylation on the metastatic characters in PDA. Mechanistic studies linking aberrant DNA methylation to the aggressive behavior of PDA in both in vitro and in vivo contexts are necessary to better elucidate the role of DNA methylation in PDA metastasis.

## 7. Therapeutic Implications of Epigenetic Regulators

Currently, there is a lack of effective chemotherapies and targeted therapies for late-stage PDA patients who make up most of the PDA patient population. The inability to identify a recurrent genetic mutation driving PDA metastasis suggests that epigenetic alterations are especially important for the tumor-to-metastasis transition and that targeting epigenetic regulators may be an effective strategy for treating late-stage patients. Fortunately, many small-molecule inhibitors of epigenetic regulators, such as histone methyltransferases, histone deacetylases (HDACs), and bromodomain and extra-terminal (BET) acetylation readers, have already been developed and have seen success as a treatment for several diseases [68]. In PDA clinical trials, these inhibitors were largely disappointing as monotherapies and have since been investigated in combination therapies with chemotherapeutic agents or other targeted therapies [69]. Unfortunately, combination therapies involving inhibitors of epigenetic regulators have shown mixed results in PDA clinical trials, highlighting a need to better understand the molecular mechanisms and synthetic lethal interactions that enable their effectiveness in preclinical studies [69].

In addition to being a potential therapeutic avenue for treating aggressive and late-stage PDA, epigenetic modifications can also be used as prognostic and/or predictive biomarkers for PDA. DNA methylation is especially promising for this purpose as circulating tumor cell-free DNA (cfDNA) in the bloodstream can be used to non-invasively identify abnormal DNA methylation patterns in tumors of PDA patients [70]. In fact, a method to detect pancreatic cancer by assessing five DNA methylation markers in cfDNA along with *KRAS* mutation status had 68% sensitivity and 86% specificity when tested on cfDNA samples from 47 pancreatic cancer patients and 14 normal volunteers [71]. Furthermore, patients who were successfully identified with this screen were more likely to have larger tumors and liver metastases, suggesting that this method could be especially useful for identifying late-stage PDA patients [71]. Methylation of individual gene promoters has also been shown to have prognostic value. For example, in methylation of *SPARC* differentiated early-stage PDA from pancreatitis patients, high methylation of *SPARC* and *NPTX2* is associated with late-stage or metastatic patients and worse survival outcomes [72]. Similarly, promoter methylation of *CDO1* was found to be specific to PDA tumors, positively correlated with PDA progression, and identifiable in pancreatic juice and small needle biopsy samples [73]. In addition, low cellular levels of H3K4me2, H3K9me2, and H3K18ac in PDA patient tumor cells were all independent and significant predictors of poor survival in a 140 patient clinical trial comparing fluorouracil to gemcitabine treatment [74], suggesting that histone modifications may have predictive value in PDA. Identification of other epigenetic biomarkers is a research area actively being pursued.

## 8. Conclusions

PDA has long been thought of as a disease arising and progressing from genetic mutations. More recently, it has become clear that epigenetic alterations are also important contributors to PDA progression and metastasis. While no single epigenetic regulator is commonly mutated in PDA, a growing body of evidence indicated that metastatic tissues exhibit distinct epigenetic status compared to primary tumor tissues, which might be exploited for cancer therapeutics and diagnostics. Studies have shown that aberrant expression of histone modification in the PDA epigenome induces chromatin remodeling that alters the recruitment of TFs and co-regulators, resulting in transcriptional changes that promote PDA aggressiveness in vitro and in vivo [15,16,17,35]. Similarly, aberrant DNA methylation has been shown to affect the transcriptome in a way that promotes the acquisition of metastatic characteristics. However, the specific transcriptional programs that most significantly affect PDA metastasis and the mechanisms by which they do so are still largely unknown and are active areas of investigation.

Overall, alterations to the epigenome clearly play an important role in PDA progression and metastasis, but the mechanisms by which they do so are not well understood. An improved understanding of these mechanisms will better inform the development of combination therapies targeting epigenetic regulators and prognostic biomarkers for the improved treatment of PDA patients, especially those in late stages.

## Figures and Tables

**Table 1:** Summary of reviewed genes that are related to epigenetic alterations during PDA progression and metastasis in the categories of germline PTV mutation, somatic mutation, chromatin accessibility and nucleosome remodeling, histone modification, and DNA methylation. ↓ denotes decrease and ↑ denotes increase.

Category	Gene	Molecular Function	Molecular Phenotype in PDA	Functional Phenotype in PDA	Reference
Germline PTV Mutation in Epigenetic Regulators	TET2	Dioxygenase of 5-methylcytosine, involved in demethylation of cytosines	loss of function in encoded protein	↓ patient survival	[12]
	DNMT3a	DNA methyltransferase, involved in de novo DNA methylation	loss of function in encoded protein	↓ patient survival	[12]
	ASXL1	Polycomb group protein, involved in gene transcriptional regulation and chromatin architecture maintenance	loss of function in encoded protein	↑ proliferation, ↓ patient survival	[12]
Somatic Mutation in Epigenetic Regulators	ARID1A	Chromatin remodeler, involved in chromatin remodeling and gene transcriptional regulation	loss of function in encoded protein	↑ progression, ↓ survival	[6,7,8,13,14]
	KDM6A	Lysine-specific histone demethylase, involved in promoter and enhancer activities	loss of function in encoded protein	↑ squamous identity, ↓ survival	[6,7,13,14]
Chromatin Accessibility	ZKSCAN1	Transcription factor, involved in proliferation and differentiation	↑ TF binding via open chromatin	↑ metastasis	[15]
	HNF1B	Transcription factor, involved in beta cell development in the pancreas	↓ TF binding via closed chromatin	↑ metastasis	[15]
Transcription Factor-Mediated Histone Modification	FOXA1	Transcription factor, involved in cell differentiation and chromatin remodeling	↑ enhancer activation (H3K27ac)	↑ cell growth, ↑ invasion, ↑ progression	[16]
	TP63	Transcription factor, involved in cell proliferation, differentiation, and apoptosis	↑ enhancer activation (H3K27ac)	↑ squamous identity	[17]
DNA Methylation	TFPI-2	Serine proteinase inhibitor, involved in negative regulation of pro-metastasis extracellular matrix degradation	↓ expression via hypermethylation	↑ progression, ↑ proliferation, ↑ migration	[18]
	RELN	Extracellular matrix serine protease, involved in neuronal migration	↓ expression via hypermethylation	↓ patient survival, ↑ migration, ↑ invasion, ↑ colony formation	[19]
	MET	Receptor tyrosine kinase, involved in cell survival, migration, and invasion	↑ expression via hypomethylation	↓ patient survival	[20]
	ITGA2	Integrin, involved in adhesion of cells to the extracellular matrix	↑ expression via hypomethylation	↓ patient survival	[20]

## Data Availability

Not applicable.
